# 

**Published:** 1922-08

**Authors:** 


### HOSPITAL and HEALTH

REVIEW

Established 1886 No. 1853. Vol. LXXI.

New Series. Vol. 1. No. 11 August 1922 Price Sixpence

CONTENTS.

PAGE

NOTES AND COMMENTS :—
Health in the Near East—The Multiplication of Mental Defective—The Prevention of Venereal Disease—The National Council for Combating Venereal Diseases—Fighting Insect-borne Diseases—Poisoned Furs—Training for Imbecile Children—Public Health and the House-fly—Ice Cream—Harmful Preservatives in Food — "Handy Women" — Panel Doctors' Absence — Typhoid Epidemic due to a Polluted Water Supply—The Frequency of Cancer in Old Age—Sunlight in the Prevention of Rickets—The Axe at Newcastle—The Problem of the Boarded-out London Child—A "Rat Barrack"—The Discovery of the Typhus Germ. . . . . . . . 305

THE PUBLIC HEALTH: INTERVIEWS WITH LOCAL AUTHORITIES. II.—THE COUNTY OF DURHAM 312

LUNACY REFORM.—II. . . . . . . 314

By H. J. Norman, M.B., Ch.B.

DO THE APPROVED SOCIETIES SEEK TO CONTROL THE DOCTORS? . . . . . . . . 316
By R. W. Harris.

PAGE

THE CONVERSION OF PAIL CLOSETS . . . . . 317

By A. T. Gooseman.

HOSPITAL ECONOMIES . . . . . . . . . 319

By Frank G. Hazell.

THE HOSPITAL PROBLEM . . . . . . . 322

THE COMBINED APPEAL FOR LONDON HOSPITALS . 324

NATIONAL BABY WEEK . . . . . . . . 325

INTERNATIONAL CONFERENCE ON BIRTH CONTROL . 327

INTERNATIONAL UNION AGAINST TUBERCULOSIS . . 328

HIGHLAND FOLK MEDICINE . . . . . . . 329

RECENT APPOINTMENTS . . . . . . . 330

REVIEWS . . . . . . . . . 331

HOSPITAL FINANCE . . . . . . . . 334

HOSPITAL NEWS . . . . . . . . 336

PARLIAMENTARY QUESTIONS AND ANSWERS . . . 337

CORRESPONDENCE :—
Maternity Homes: The Henry Saxon Snell Prize—A Central Fund for Provincial Hospitals—Home Nursing for Insured Persons . . . . . 338

